# Dental black plaque: metagenomic characterization and comparative analysis with white-plaque

**DOI:** 10.1038/s41598-020-72460-2

**Published:** 2020-09-29

**Authors:** Verónica Veses, Pedro González-Torres, Belén Carbonetto, Mª del Mar Jovani-Sancho, Raquel González-Martínez, Isidoro Cortell-Ballester, Chirag C. Sheth

**Affiliations:** 1 Department of Biomedical Sciences, Faculty of Health Sciences, Universidad Cardenal Herrera-CEU, CEU Universities, 46113 Moncada, Valencia, Spain; 2 Microomics Systems S.L, Barcelona, Spain; 3 Department of Dentistry, Faculty of Health Sciences, Universidad Cardenal Herrera-CEU, CEU Universities, 46113 Moncada, Valencia, Spain; 4 Department of Medicine, Faculty of Health Sciences, Universidad Cardenal Herrera-CEU-, CEU Universities, 46113 Moncada, Valencia, Spain

**Keywords:** Bacteria, Microbial communities, Next-generation sequencing, Dental diseases, Microbial ecology

## Abstract

Extrinsic black dental staining is an external dental discoloration of bacterial origin, considered a special form of dental plaque. Currently, there is no definitive therapeutic option for eliminating black stain. This study employed 16S rRNA metagenomics to analyze black stain and white-plaque samples from 27 adult volunteers. Study objectives were to: describe the microbial diversity of adult black stain samples; characterize their taxonomic profile; compare the microbiomes of black stain versus white-plaque from adult volunteers and propose a functional map of the black stain microbiome using PICRUSt2. The black stain microbiome was poorer in species diversity as compared to white-plaque. The five most abundant genera in black stain were *Capnocytophaga*, *Leptotrichia*, *Fusobacterium*, *Corynebacterium* and *Streptococcus*. Functional analysis of microbial species revealed conserved and consistent clustering of functional pathways within and between black stain and white-plaque microbiomes. We describe enrichment of heme biosynthetic pathways in black stain. Our results suggest that the dysbiosis in black stain resembles “orally healthy” communities. The increased abundance of heme biosynthetic pathways suggests that heme-dependent iron sequestration and subsequent metabolism are key for black stain formation. Further research should decipher the regulation of heme biosynthetic genes and characterize the temporal sequence leading to colonization and dysbiosis.

## Introduction

The oral microbiome is the second most complex after the gut microbiome, with an estimated 687 predominant bacterial species^1,2^. Specific microbial communities can be found for each oral cavity site: tongue, buccal mucosa, tooth surfaces, soft and hard palate, tonsils and lip vestibule. In addition, the salivary microbiome is considered as comprising of a mixture of all those sites’ microbiomes^3^. Healthy dental plaque on tooth surfaces and gingival margin is considered as being amongst the most stable and diverse microbial communities in the mouth^4^.

Dental plaque has been clinically defined as “the microbial community that develops as a structurally- and functionally-organized biofilm on the tooth surface, embedded in a matrix of polymers of bacterial and host salivary origin”^5^. Recent advances in high-throughput sequencing and metagenomics have permitted a deeper understanding of the microbial the composition of dental plaque in healthy individuals. Ten leading (abundance > 1%) genera have been shown to be commonly found in supragingival dental plaque: *Streptococcus*, *Veillonella*, *Granulicatella*, *Fusobacterium*, *Neisseria*, *Campylobacter*, *Gemella*, *Abiotrophia*, *Selenomonas* and *Capnocytophaga*^6^. When sub- and supra-gingival plaque microbiomes are analyzed jointly, eleven genera with a relative abundance over 2% have been described: *Streptococcus*, *Veillonella*, *Prevotella*, *Neisseria*, *Fusobacterium*, *Actinomyces*, *Leptotrichia*, *Corynebacterium*, *Capnocytophaga*, *Rothia* and *Porphyromonas*^7^.

It is well established that changes in the composition and structure of the oral microbiome are related to diseases, such as caries, periodontal disease or odontogenic infections^3,8^. According to the “keystone pathogen” hypothesis by Hajishengallis, oral diseases arise as a consequence of disruptions in the oral microbiome, initiated by low-abundant pathogens, which finally drive an ecological imbalance or dysbiosis^9^. It was seen than when these ecological shifts occur in dental plaque they lead to caries^10^. In this context, several recent studies have focused on the study of microbiome changes in dental plaque in individuals with caries^6,11,12^.

We hypothesize that similar changes may promote the formation of extrinsic black dental staining (BS). The exact origin and nature of these microbial changes currently remain elusive^13^. BS is considered a special type of dental plaque, formed by microorganisms within an organic matrix^14^. BS is characterized by the formation of dots or black lines distributed in parallel to the gingival margin of teeth and firmly attached to the enamel^15^. Children are mainly affected, but it can also be observed in adults, with a prevalence ranging from 1 to 20%^16^. Currently, there is no long-term therapeutic solution for patients with BS, who commonly undergo repeated rounds of dental prophylactic cleaning in order to maintain a stain-free oral cavity. The observed dark pigment in BS has been described by some authors, as a black insoluble ferric compound formed by bacteria in the dental plaque^17^. This has been recently confirmed by Zhang et al. who observed higher iron levels in BS patients than in white plaque patients using ICP-MS analysis^18^. A microbiological analysis of BS samples from children’s teeth highlighted the presence and importance of chromogenic bacteria such as *Prevotella melaninogenica, Actinomyces israelii* and *Actinomyces naeslundii*^19^. Application of PCR techniques have contributed to broaden the knowledge regarding the bacterial composition of BS, nevertheless results remain controversial. Saba et al. found *Porphyromonas gingivalis*, *P. melaninogenica*, *Actinomyces spp*, and *Aggregatibacter actinomycemcomitans* to be prevalent in BS, while Li et al. revealed a different core community composed of *A. actinomycemcomitans*, *Prevotella intermedia*, *Cardiobacterium spp*, *Haemophilus spp*, *Corynebacterium spp*, *Tannerella spp* and *Treponema spp* as the main species^20,21^.

To our knowledge, currently there are only two publications presenting BS microbiome analysis based on metagenomics approaches. Significantly, both studies used dental plaque samples from pediatric patients and reported few or no differences in community diversity between BS and non-BS samples. Differences in taxa relative abundances were reported, with *Actinomyces naeslundii* found to be more abundant in BS samples and *Candidate_division_TM7* more abundant in non-BS samples^22^. Moreover, *Leptotrichia* and *Fusobacterium* were suggested to play an important role in the formation of pigment in primary dentition^23^.

The main objectives of this study are to: describe the microbial diversity of adult BS; characterize the microbial community found in adult BS; compare the taxonomic profiles of adult BS and white-plaque; and develop a functional map of the adult BS microbiome with a view to advancing the knowledge related to microbial dysbiosis and BS in the oral cavity. To our knowledge, this is the first study to investigate the dysbiosis hypothesis in adult patients with BS using a metagenomics approaches.

## Results

### Demographic characteristics of the study volunteers

**Figure 1:**
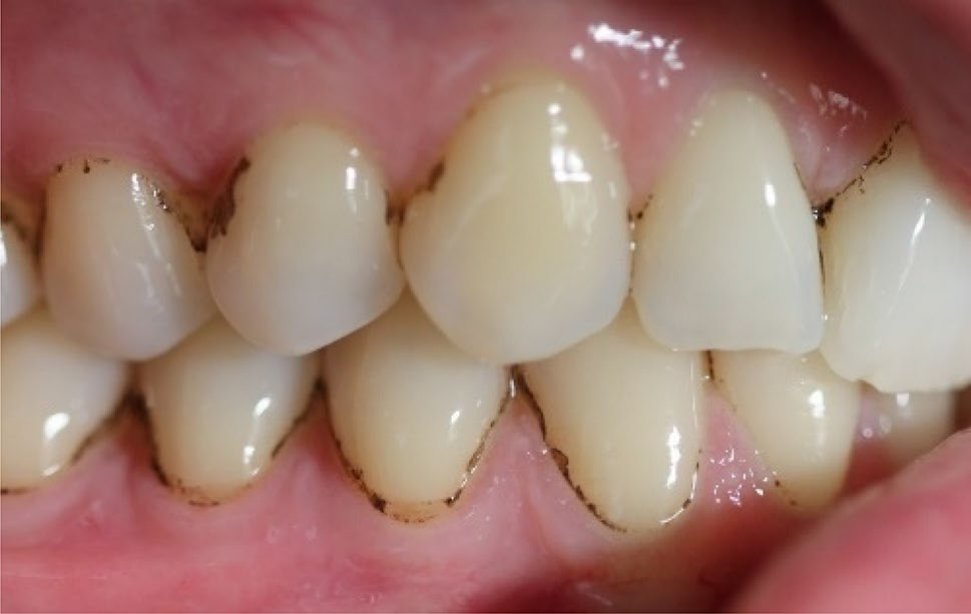
Dental black stain in an adult patient. BS appears as dots or black lines distributed in parallel to the gingival margin of teeth in the cervical third.

A total of 27 adult volunteers participated in the study. All participants were free of caries, gingivitis or periodontitis, with a DMFT index of < 4.4^24^. Nine were orally healthy individuals whilst 18 had BS in two or more teeth (Fig. 1). The mean age in the control group was (42.9 ± 10.1) whereas the mean age of the patients with BS was (43.8 ± 15.8) (Table 1).

**Table 1:** Demographic characteristics of the participants, including the sex distribution, mean age and total number of stained teeth.

	Control	Black Stain
Male % (n)	22.2% (2)	38.9% (7)
Female % (n)	77.7% (7)	61.1% (11)
Mean age in years (SD)	42.9 ± 10.1	43.8 ± 15.8

### Diversity of the BS microbiome

**Figure 2:**
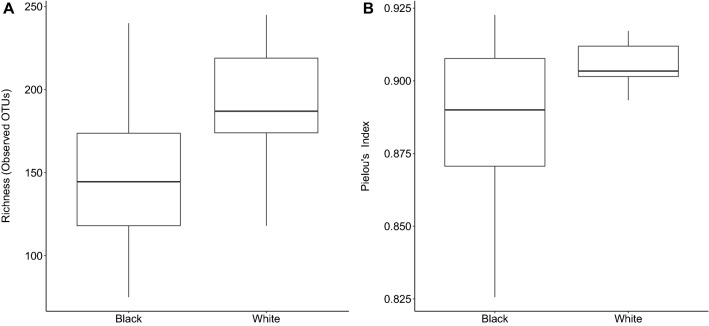
Alpha diversity analysis, comparison between BS and white-plaque samples. (**A**) Diversity richness expressed as the number of observed OTUs (i.e. phylotypes) and (**B**) Pielou’s evenness index.

Amplicon sequencing results revealed higher microbial richness in white-plaque biofilm samples as compared to BS (*p* = 0.01 Fig. 2A). However, we observed no significant difference in Pielou’s evenness index between BS and white-plaque samples (Fig. 2B).

**Figure 3:**
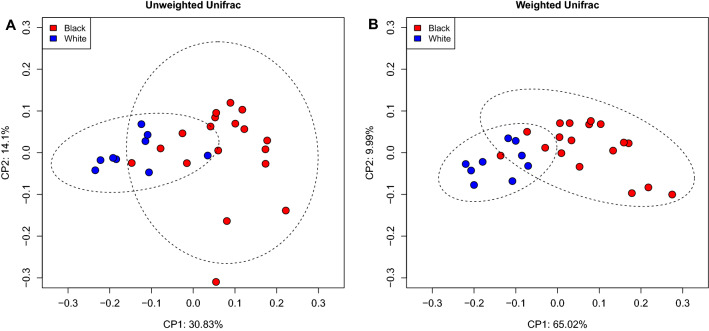
Beta diversity analysis. Principal coordinate analysis based on unweighted Unifrac (**A**) and weighted Unifrac (**B**) distance matrices. The differences in community structure between BS and white-plaque samples were confirmed by Permanova test (*P* = 0.001).

Beta diversity analyses based on Unifrac phylogenetic-based distances showed differences in the structure of microbial communities between BS and white-plaque biofilms (Fig. 3). Differences were observed for both unweighted (Fig. 3A) and weighted Unifrac (Fig. 3B) distances (Permanova *p* = 0.001 for both distances) revealing that black and white biofilms differ in quality (i.e. presence/absence) and in abundance of phyloypes. Jaccard qualitative distance and Bray Curtis quantitative distance were also used, results also showed differences between BS and white-plaque samples (Supplementary Fig. 1).

### Taxonomic composition of the BS microbiome

**Figure 4:**
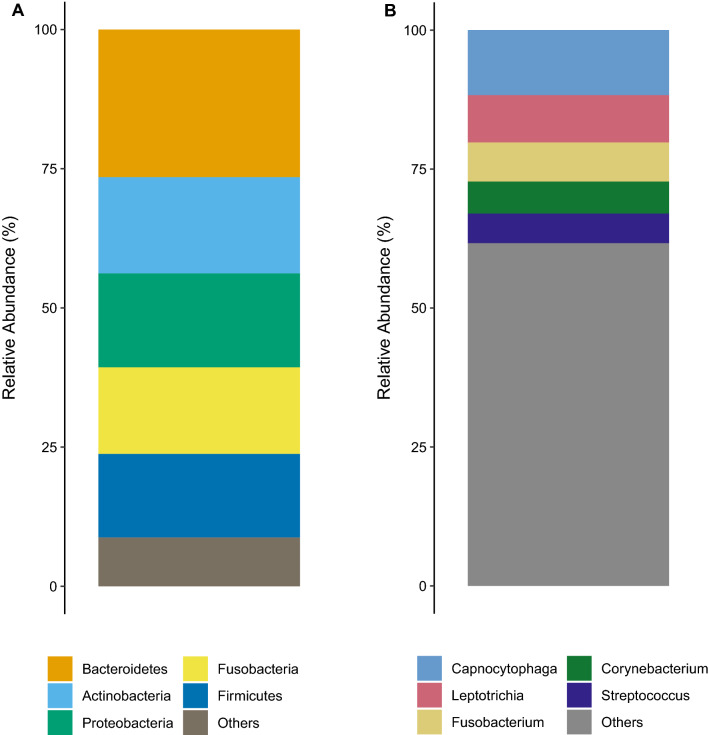
Top five most abundant phyla (**A**) and genera (**B**) in the BS biofilm. Average relative abundances are shown.

Eleven bacterial phyla were detected in BS samples. The top five most abundant phyla were found to be *Bacteroidetes* 26.5% [95% CI (24.9–28.1)], *Actinobacteria* 17.4% [95%CI (14.2–20.3)], *Proteobacteria* 16.9% [95%CI (14.0–19.8)], *Fusobacteria* 15.6% [95%CI (13.1–18.0)] and *Firmicutes* 15.0% 95%CI (13.2–16.8)] (Fig. 4A). These 11 phyla constituted 91.4% of the average relative abundance of the reads (Fig. 4A). A total of 109 distinct genera were observed in BS samples. The five most abundant genera, constituting 38.34% average relative abundance were found to be *Capnocytophaga* 11.7% [95%CI (10.0–13.4)], *Leptotrichia* 8.5% [95%CI (6.8–10.3)], *Fusobacterium* 7.1% [95%CI (6.2–7.9)], *Corynebacterium* 5.7% [95%CI (4.7–6.8)], and *Streptococcus* 5.3% [95%CI (4.0–6.7)] (Fig. 4B).

### Comparison of the taxonomic profiles of BS and white-plaque

**Figure 5:**
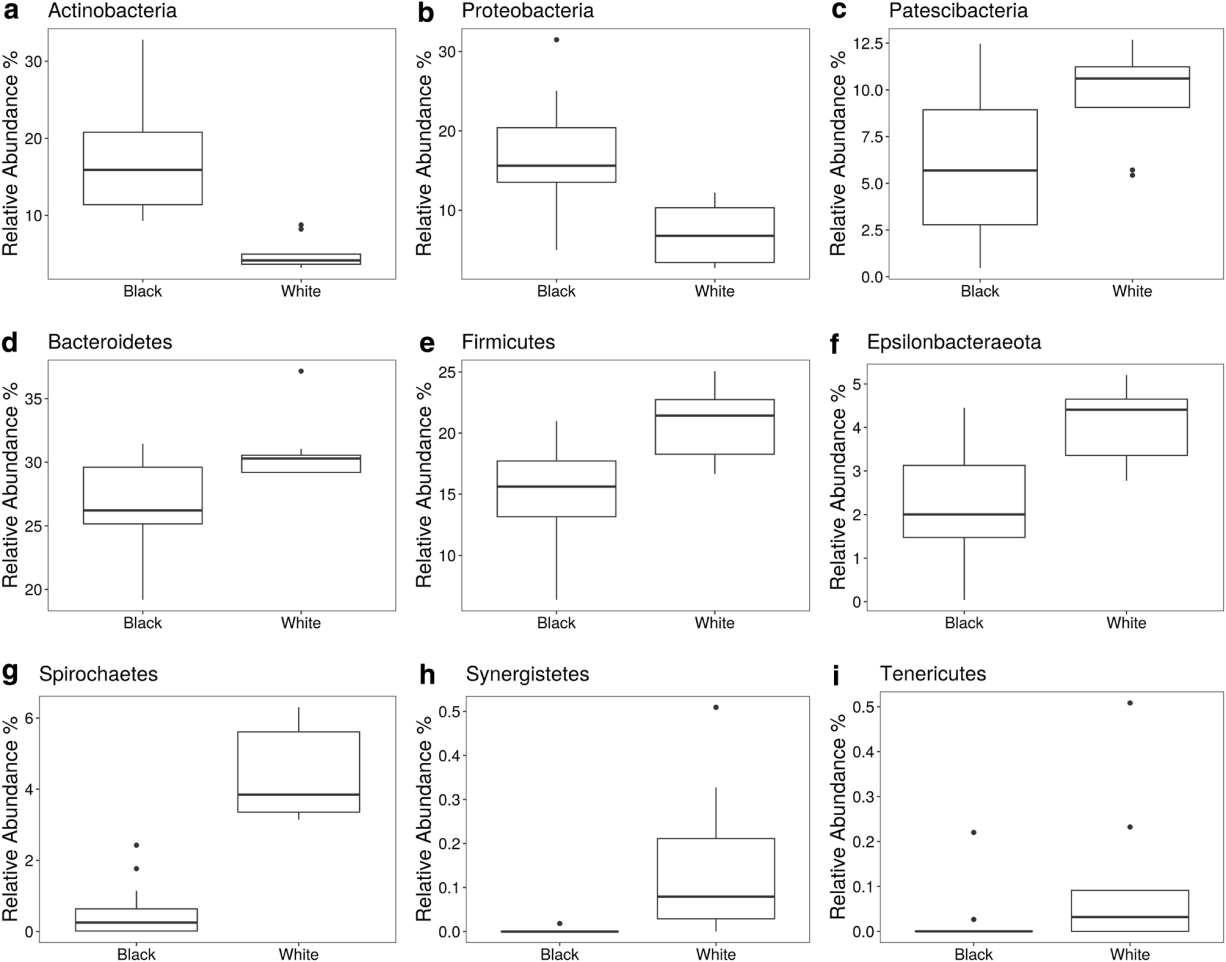
Differences in abundance of key phyla between BS (black) and white-plaque (white) samples.

A comparative analysis between BS and white-plaque samples was carried out. Differences in relative abundances of phyla between BS and white plaque samples were characterized using a Kruskal Wallis non-parametric test. Of the top 5 phyla in terms of abundance, we found *Actinobacteria*, and *Proteobacteria* to be more abundant in BS samples than in white plaque samples (Fig. 5a,b), whilst *Bacteriodetes and Firmicutes* were more abundant in white plaque samples as compared to BS samples (Fig. 5d,e)*.* Amongst the bacteria of lesser abundance, we found that the relative abundances of *Patescibacteria, Epsilonbaceraeota*, *Spirochaetes*, *Synergistetes* and *Tenericutes* were found to be higher in white plaque samples as compared to BS samples (Fig. 5c,f–i), Table 2).

**Table 2:** Difference in relative abundance of phyla in BS versus white-plaque samples.

Phylum	Fold difference on average relative abundances (black:white)	*p*-value
**Actinobacteria**	**3.46**	**0.0000002**
Bacteriodetes	0.86	0.0021
Epsilonbaceraeota	0.57	0.00064
Firmicutes	0.73	0.00082
Patescibacteria	0.61	0.017
**Proteobacteria**	**2.34**	**0.0000027**
Spirochaetes	0.12	0.00000017
Synergistetes	0.01	0.000000025
Tenericutes	0.13	0.0017

Over represented taxa in BS samples are shown in bold. *p* values after Kruskal Wallis test with FDR correction.

**Figure 6:**
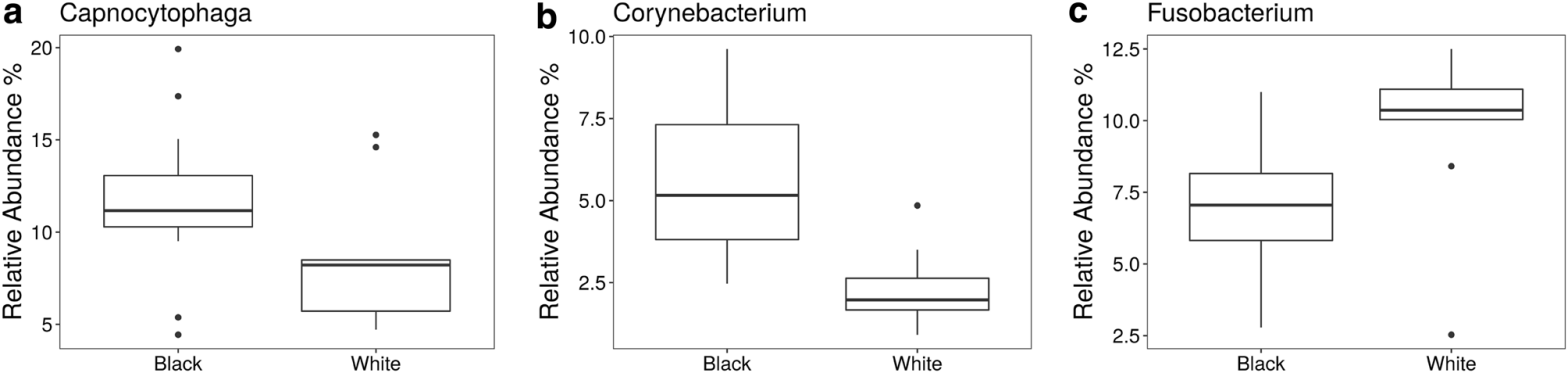
Differences in abundance of key genera between BS (black) and white-plaque (white) samples.

Overall, at the genus level, we observed 42 microbial genera with differential abundance between BS and white-plaque samples (Supplementary Table 1). Of the top 5 most abundant genera identified in BS, comparative analysis with white-plaque samples showed that *Capnocytophaga* and *Corynebacterium* were found in higher relative abundance in BS samples (Fig. 6a,b) while *Fusobacterium* was present in higher abundance in white-plaque samples (Fig. 6c).

### Functional analysis of the BS microbiome

**Figure 7:**
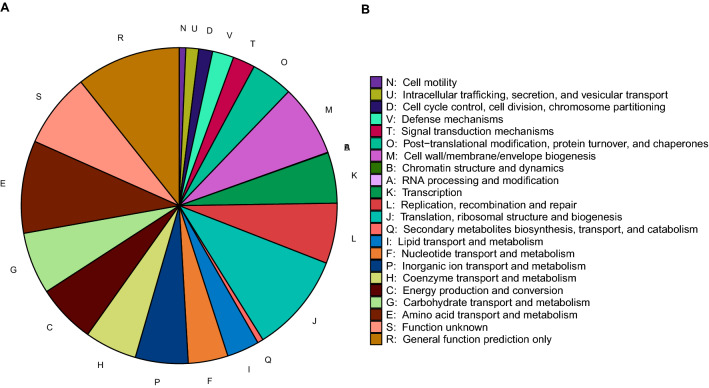
Average relative abundances (%) of COG functional categories in the BS microbiome.

The metagenomic analysis of the BS and white-plaque samples allowed a deeper analysis of the potential functional composition of the microbiome using PICRUSt2 (Fig. 7). The functional analysis was based on two annotations: the Clusters of Orthologous Groups of proteins (COG) and the Enzyme Commission numbers (EC) to further predict Metacyc reactions and infer pathway abundances.

A comparison of the predicted metagenomes of BS and white-plaque samples revealed the differential abundance of 2.222 COGs (Supplementary Table 2) and 183 Metacyc pathways (Supplementary Table 3). Hierarchical clustering of samples according to the abundance of COG and Metacyc pathways were observed (Supplementary Figs. 2 and 3). These results demonstrate conserved and consistent clustering of functional pathways within and between BS and white-plaque microbiomes.

The COG functional groups detected in BS, in rank order were “metabolism”, “information storage and processing”, followed by “cellular processes and signaling” (Fig. 7). Specifically, the following top 5 known pathways were found in greater relative abundance in BS; “translation, ribosomal structure and biogenesis”, “amino acid transport and metabolism”, “Cell wall/membrane/envelope biogenesis”, “carbohydrate transport and metabolism” and finally “Replication, recombination and repair”.

**Figure 8:**
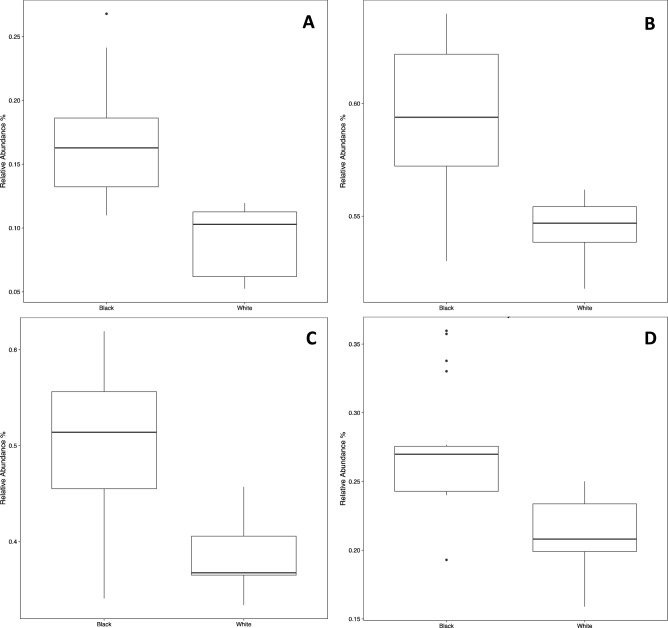
Box-and-whisker plots showing the relative abundances of four pathways statistically significantly enriched in BS samples; (**A**) the superpathway of heme biosynthesis from uroporphyrinogen-III (PWY0-1415), (**B**) tetrapyrrole biosynthesis- I from glutamate (PWY-5188), (**C**) anaerobic heme biosynthesis (HEMESYN2-PWY), and (**D**) aerobic heme biosynthesis I (HEME-BIOSYNTHESIS-II).

Amongst the 183 Metacyc pathways identified when comparing BS with white-plaque samples, we observe a statistically significant enrichment in the abundance of heme biosynthetic pathways in BS samples (Fig. 8). The relative abundances of the superpathway of heme biosynthesis from uroporphyrinogen-III (PWY0-1415; Fig. 8A), tetrapyrrole biosynthesis-I from glutamate (PWY-5188; Fig. 8B), anaerobic heme biosynthesis (HEMESYN2-PWY; Fig. 8C), and aerobic heme biosynthesis I (HEME-BIOSYNTHESIS-II; Fig. 8D) pathways were all found to be higher in BS as compared with white-plaque samples. The mycolyl-arabinogalactan-peptidoglycan complex biosynthesis pathway was also found in greater abundance in BS (data not shown). This is a pathway involved in the synthesis of cell wall components in *Corynebacterium*, supporting the increased abundance of the genera detected in BS samples during the taxonomic characterization analysis. These data demonstrate the pathways differentially abundant in BS as compared to white-plaque, which suggest a significant change in the overall microbial community functions between the two sample types.

## Discussion

This study aimed to employ metagenomics to characterize the microbiome associated with supragingival dental plaque in adult patients with BS, as compared with white-plaque. We also attempted to develop a functional map of the bulk microbial metabolism through PICRUSt2 analysis. Thus far, existing investigations have centered on BS samples taken from pediatric patients. This is the first study characterizing the microbial community in BS and comparing it to white-plaque in adult volunteers.

With regard to the alpha diversity, we found that BS had lower species richness and evenness as compared to white-plaque. The differences were statistically significant (Fig. 2). Li et al., using salivary samples from pediatric volunteers, described results similar to those in the current study (lower diversity in BS versus control), however there were no statistically significant differences between samples of BS and white-plaque^23^. Chen et al. found no differences in alpha diversity between BS and white-plaque samples from pediatric patients^22^. The difference in results between studies using samples from pediatric volunteers and our study using adult volunteers demonstrates a clear distinction in the composition and diversity of the oral microbiome. Whilst pediatric samples demonstrate little or no difference in species diversity in plaque samples and richness, our results from adult patients suggest that a strong dysbiosis may develop over time in these patients.

We showed that, in comparison with white-plaque, patients with BS harbor a statistically significantly different microbial community in terms of presence of individual species and their relative abundances (Fig. 3). The difference between BS and white-plaque demonstrates conclusively the presence of a dysbiosis. There is currently no data available for comparison regarding the beta diversity of BS compared to white-plaque in either adult or pediatric samples.

We were able to detect a total of 11 distinct bacterial phyla (Fig. 4A) and 109 different genera (Fig. 4B) within BS samples. Previous studies highlighted the presence of *Prevotella* and *Actinomyces* species, which were not amongst the top 5 genera identified in our study. It should be noted that this research was carried out using culture-based techniques, which have since been superceded by modern molecular methodology^19^. More recently Saba et al. and Li et al. used PCR and next-generation sequencing-based detection to identify the composition of BS in pediatric samples. Only one genus was found in common with the current study (*Corynebacterium*), suggesting that the composition of pediatric and adult BS is significantly different^20,21^. It is notable that there was only one genus (*Aggregatibacter*) in common between the Saba and Li studies.

Interestingly, genera such as *Capnocytophaga* and *Corynebacterium* are commonly found in greater abundance in individuals with healthy oral cavities (lower caries experience). Overall, all members are commonly found in the oral cavity, however, are not amongst the most common. There are significant differences amongst the top 5 bacterial phyla when comparing BS with white-plaque samples (Fig. 5). *Actinobacteria* and *Proteobacteria* are enriched in BS as compared to white-plaque, whilst *Bacteroides* and *Firmicutes* are found in greater abundance in white-plaque as compared to BS in adult volunteers. In pediatric samples, Chen et al. obtained similar results to our study, although the differences were not statistically significant^22^. Likewise, Li et al. showed an increase in abundance of *Actinobacteria* in plaque samples from children with black stain, however did not describe significant differences in *Proteobacteria*, *Bacteriodes* or *Firmicutes*^23^. This could be due to differences between the study populations, since the participants in the Chen and Li studies were pediatric, whilst our research included adult participants with a mean age of 43.8 years.

At the genus level (Fig. 6), data published by Chen et al. contradicts our results, as they show a relatively higher abundance (although not statistically significant) of *Capnocytophaga* and *Corynebacterium* in white-plaque as compared to BS samples. They coincide with our results in that they observed an enrichment of *Fusobacterium* in white-plaque samples^22^. Li et al. on the other hand, coincide partly with our data, showing an increased abundance of *Capnocytophaga* and *Corynebacterium* in saliva samples of children with black stain. They did not describe statistically significant differences in the relative abundance of *Fusobacterium*^23^. It is important to bear in mind that the existing studies have been carried out on pediatric samples and therefore any variations must be evaluated in the context of the age difference.

Earlier research has linked the increased presence of *Corynebacterium*, *Capnocytophaga*, *Fusobacterium* and *Leptotrichia* amongst others, with improved oral health and lower caries experience^25^. The top 5 phyla identified as part of the BS microbiome in this study coincide completely with the healthy “core microbiome” described by Zaura et al., although the rank order of relative abundances vary between the two studies. This suggests that the dysbiosis represented by BS may play a protective role against caries^26^. Similar results were published by Belstrøm et al. who compared saliva from healthy patients with samples active caries and periodontitis. *Bacteriodes*, *Actinobacteria*, and *Fusobacteria* were all found to be more abundant in saliva from healthy patients as compared to those with oral disease^27^. When comparing the results of our study with supragingival plaque sample analysis of patients with periodontitis, we observe that *Bacteroides* and *Fusobacteria* are enriched in both BS and periodontitis patients, although we concurrently observe an increased abundance of Firmicutes and Actinobacteria in BS, both of which are associated with healthy oral microbiomes^28^.

The progression of the oral dysbiosis leading to BS is as yet unclear, however we propose a mechanism based on the ecological plaque hypothesis in which local oral environmental conditions promote the growth and enrichment of key species leading whilst inhibiting the accumulation of others^29^. Our study shows that BS has a composition resembling mature dental plaque biofilm, with several key differences; the reduced abundances of *Porphyromonas* and *Prevotella*^30^. This could explain the lack of gingival inflammation and the generally better oral health observed in patients with BS as compared to white-plaque. We find a relative decrease in abundance of “yellow complex” and “red complex” bacterial species, both of which are associated with oral disease states, with a concurrent increase in species associated with oral health^31^. Our findings suggest that the microbial community found in BS acts as a driver for a supragingival biofilm characterized by low oral inflammation and thus promoting lower periodontal disease. Previous research from our group has shown that individuals with BS have higher salivary pH, thus confirming the promotion of an anti-caries environment in the oral cavity^16^.

Our PICRUSt2 analysis reveals significant alterations in the abundances of functional pathways between BS and white-plaque samples (Figs. 7 and 8). We observed an enrichment of heme-biosynthesis pathways, supporting the theory that the black stain may be the black insoluble ferric deposits described by Reid et al.^17^. High iron levels were detected in black stain by ICP-MS analysis^18^. Preliminary ICP-MS data from our group suggest that BS is also associated with high iron levels in supragingival BS (data not shown). It is not possible to determine the temporal sequence of events resulting in the formation of BS from the current study. The heme and iron compound biosynthesis may precede the formation of BS or constitute an integral part of the development process as a result of the actions of the predominant genera present.

Microbiome analysis of BS from adult volunteers reveals a profound dysbiosis when compared with white-plaque samples. This dysbiosis is not observed in pediatric patients and there is a lack of comparative studies using adult volunteers. The microbiome composition in BS aligns with a “orally healthy” community as identified in previous studies. The temporal sequence resulting in dysbiosis (colonizers and enrichment) and the pathway employed for the generation and deposition of black pigment remains elusive. PICRUSt2 analysis confirms an increased relative abundance of heme biosynthetic pathways in BS, which, when combined with evidence demonstrating increased iron abundance in BS, suggest that heme-dependent iron sequestration and subsequent metabolism may play a role in BS generation. Further research could be targeted at confirming the upregulation of heme biosynthetic genes in BS, identifying the microbial species responsible for BS and the temporal sequence leading to colonization and dysbiosis. It remains to be determined whether the heme biosynthesis pathway would constitute a valid therapeutic target for the treatment of BS.

## Materials and methods

### Dental plaque sample collection

Fresh supragingival dental plaque samples (n = 27 [18 black plaque samples and 9 white plaque samples]) were obtained from adult patients attending the university dental clinic at CEU Cardenal Herrera University. The power of a one-sided test, under the assumptions that the responses are distributed according to a Laplace parent distribution, with 18 participants in one group and 9 in the other, α = 0.05 and an effect size, *d* = 1, was calculated, and found to be 90.3%, indicating that the number of volunteers participating in the study was statistically significant. Volunteers were selected on the basis of the inclusion and exclusion criteria shown in Table 3. Plaque samples were collected by MDMJ, RGM and ICB, using plastic dental curettes and following standard plaque sample removal procedures. All samples were obtained after informed consent, and this research was reviewed and approved by the University Ethics Committee. Samples were stored at 80 °C until further processing.

**Table 3:** Inclusion and exclusion criteria for participants.

Inclusion criteria	Exclusion criteria
*Black plaque-positive subjects*: Having at least 2 teeth with black stain, with a score of > 2 (according to the scale published by Gasparetto ^32^), negative for other extrinsic discoloration	Presence of caries, gingivitis or periodontitis
*White plaque positive subjects:* Negative for black and other extrinsic discoloration	Any systemic disease
*All subjects*: 18–65 years of age	Having received antibiotics during 3 months prior to the start of the study
	Having received a dental cleaning during 3 months prior to the start of the study

### DNA extraction and PCR amplification

DNA Extraction and Library preparation were performed by Microomics Systems S.L. DNA was extracted from plaque samples using the DNeasy PowerLyzer PowerSoil Kit (Qiagen) following manufacturer’s instructions. The extraction tubes were agitated twice using Tissue lyser II (Qiagen) at 30 Hz/s for 5 min. A negative control containing no template DNA was included from purification steps. As a positive control for downstream procedures, ZymoBIOMICS Microbial Community DNA Standard (ZymoResearch) was used. This “mock community” comprises a proportioned mixture of bacterial and fungi DNA from selected species, designed to deliver reproducible and predictable results upon amplification. Samples were amplified using specific primers for the V3-V4 regions of the 16S rRNA gene . (5′-TCGTCGGCAGCGTCAGATGTGTATAAGAGACAGCCTACGGGNGGCWGCAG-3′) and V3-V4-Reverse (5′GTCTCGTGGGCTCGGAGATGTGTATAAGAGACAGGACTACHVGGGTATCTAATCC-3′). The PCR was performed in 10-μL final volume with 0.2-μM primer concentration. The PCR included: 3 min at 95 °C (initial denaturation) followed by 25 cycles: 30 s at 95 °C, 30 s at 55 °C, and 30 s at 72 °C, and a final elongation step of 5 min at 72 °C. PCR products were purified using AMPure XP beads (Beckman Coulter, Nyon, Switzerland) with a 0.9 × ratio according to the manufacturer’s instructions. PCR products were eluted from the magnetic beads with 30 μL of Milli-Q water. The above-described primers contain overhangs allowing the addition of full-length Nextera barcoded adapters for multiplex sequencing in a second PCR step, resulting in sequencing ready libraries with approximately 450 bp insert sizes. In brief, 5 μL of the first PCR purified product were used as the template for a second PCR with Nextera XT v2 adaptor primers in a final volume of 30 μL using the same PCR mix and thermal profile as for the first PCR but with only 8 cycles. 25 μL of the second PCR product were purified with SequalPrep normalization kit (Invitrogen, ThermoFisher Scientific, Waltham, MA, USA), according to the manufacturer’s protocol. Libraries were eluted in 20 μL final volume and pooled for sequencing.

Sequencing was performed in an Illumina MiSeq with 2 × 300 bp reads using v3 chemistry with a loading concentration of 10 pM. In all cases, 15% of PhIX control.

libraries was spiked in to increase the diversity of the sequenced sample. Negative controls included sample collection buffer, DNA extraction, and PCR amplification steps, PRC products after both PCR steps were visualized using an electrophoresis gel (1.5% agarose) with SYBR Safe (Applied Biosystems, ThermoFisher Scientific, Waltham, MA, USA). No visible bands were observed.

### Sequence and analysis

Raw demultiplexed forward and reverse reads were processed using the following methods and pipelines as implemented in QIIME2 version 2019.4 with default parameters unless stated^33^. DADA2 was used for quality filtering, denoising, pair- end merging and amplicon sequence variant calling (ASV, i.e. phylotypes) using qiime dada2 denoise-paired method^34^. Q20 was used as quality threshold to define read sizes for trimming before merging (parameters: -p-trunc-len-f and -p-trunc-len-r). Reads were truncated at the position when the 75th percentile Phred score felt below Q20: 273 nt for Forward and 225 nt for Reverse reads. After quality filtering steps, average sample size was 28,611 reads (min: 18,887 reads, max: 47,572 reads) and 2,653 phylotypes were detected. ASVs were aligned using the qiime alignment mafft method^35^. The alignment was used to create a tree and to calculate phylogenetic relations between ASVs using qiime phylogeny fasttree method^36^. ASV tables were subsampled without replacement in order to even sample sizes for diversity analysis using qiime diversity core-metrics-phylogenetic pipeline. The smallest sample size was chosen for subsampling . Jaccrad, Bary Curtis and unweighted and weighted Unifrac distances were calculated to compare community structure^37^. The following alpha diversity metrics were calculated: observed OTU number (i.e. richness) and Pielou’s evenness index. Taxonomic assignment of ASVs was performed using a Bayesian Classifier trained with Silva database (i.e. 99% OTUs database) using the qiime feature-classifier classify-sklearn method^38^. Phylotypes were filtered to discard contaminant Eukariota DNA-derived amplicons using Blast against the mentioned database with a 90% identity cutoff.

Differential abundance of taxa was tested using Kruskal Wallis non-parametric test. FDR Benjamini–Hochberg correction was used to correct for multiple comparisons. Alpha diversity comparisons were performed using Kruskal–Wallis non-parametric test. Unifrac, Jaccard and Bray Curtis distance matrices and ASV count tables were used to calculate principal coordinates and construct ordination plots. The significance of groups (i.e. black vs. White plaque samples) in community structure was tested using Permanova. Permdisp test was used to identify location vs. dispersion effects⁠. Significant threshold was set at *p* ≤ 0.05. ‘BiodiversityR’ version 2.11-1, ‘PMCMR’ version 4.3 and ‘vegan’ version 2.5-5 packages of the R software package version 36.6.0 (www.R-project.org) were used.

Potential functional profiles for sequenced samples were predicted using PICRUSt2^39^. In summary, phylotypes were placed into a reference tree containing 20,000 full 16S rRNA genes from prokaryotic genomes in the Integrated Microbial Genomes (IMG) database.

Functional annotation of these genomes was based on Clusters of Orthologous Groups of proteins (COG) and the Enzyme Commission numbers (EC) databases. MetaCyc ontology was used for inference of pathway abundances using MinPath^40^. In order to infer Metacyc pathways, EC numbers were first regrouped to MetaCyc reactions. Pathway abundances were calculated as the harmonic mean of the key reaction abundances in each sample. To infer the abudance of each gene family per sample: the abundances of phylotypes were corrected by their 16S rRNA gene copy number and then multiplied by their functional predictions. The analysis of differential relative abundances of Metacyc Pathways and COGs was perfomed with Kruskal Wallis test. FDR Benjamini–Hochberg correction was used to correct for multiple comparisons. Package ‘pheatmap’ version 1.0.12 of the R software package version 36.6.0 was used for hierarchical clustering and heatmap ploting.

The metagenome data have been deposited in the SRA (sequence read archive) database, belonging to NCBI (National Centre for Biotechnology Information), with accession number: PRJNA644495.

### Ethical approval

All procedures performed in studies involving human participants were in accordance with the ethical standards of the institutional and national research committee and with the 1964 Helsinki Declaration and its later amendments or comparable ethical standards. The study was approved by the Ethics Committee of CEU Cardenal Herrera University (authorization number CEI16 / 019).

### Informed consent

Informed consent was obtained from all individual participants included in the study.

## Supplementary information

Supplementary Table 3.

Supplementary Figure 1.

Supplementary Figure 2.

Supplementary Figure 3.

Supplementary Table 1.

Supplementary Table 2.

Supplementary Legends.
