# Supplementary figures and images for: Dental black plaque: metagenomic characterization and comparative analysis with white-plaque
Source: Sci Rep. 2020 Sep 29;10:15962. doi: 10.1038/s41598-020-72460-2 (PMC7525459; doi:10.1038/s41598-020-72460-2)

A

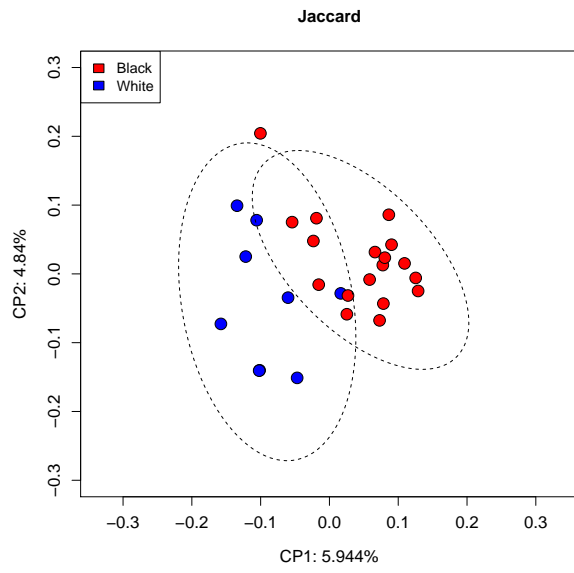

B

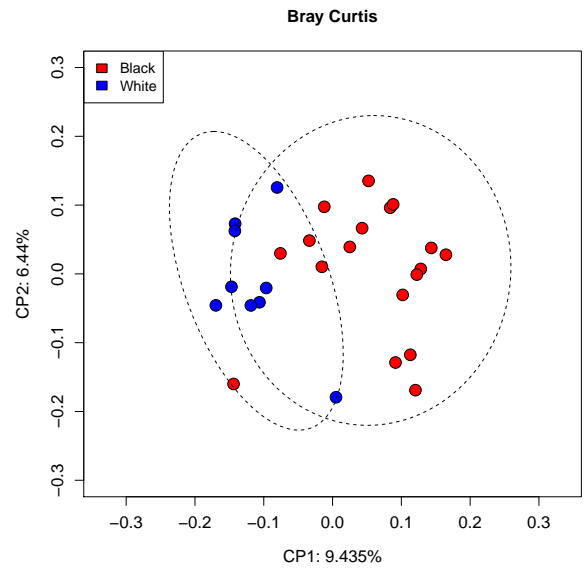

Supplement: Supplementary file 2 — Supplementary Figure 1. [file 41598_2020_72460_MOESM2_ESM.pdf]

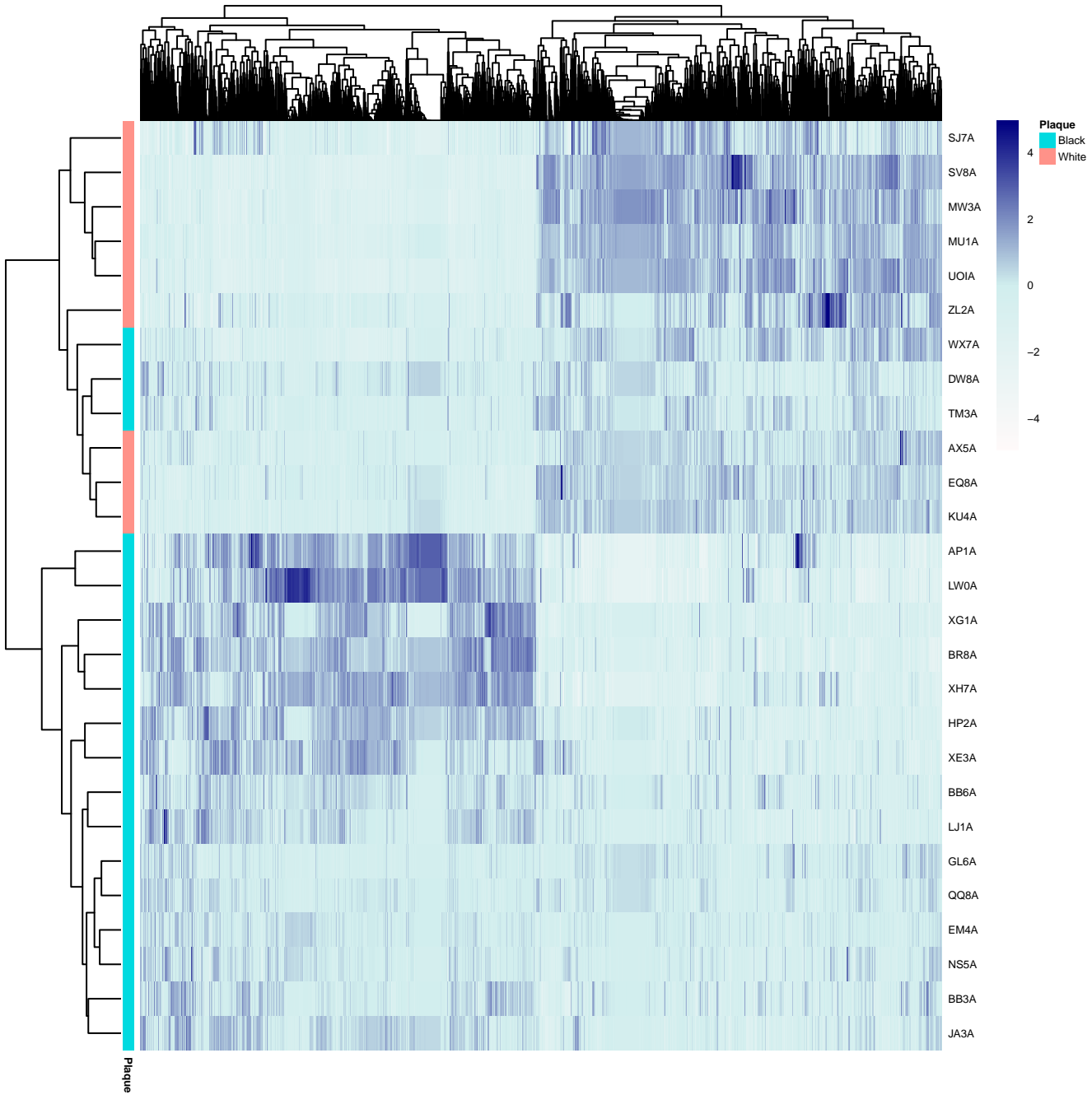

Supplement: Supplementary file 3 — Supplementary Figure 2. [file 41598_2020_72460_MOESM3_ESM.pdf]

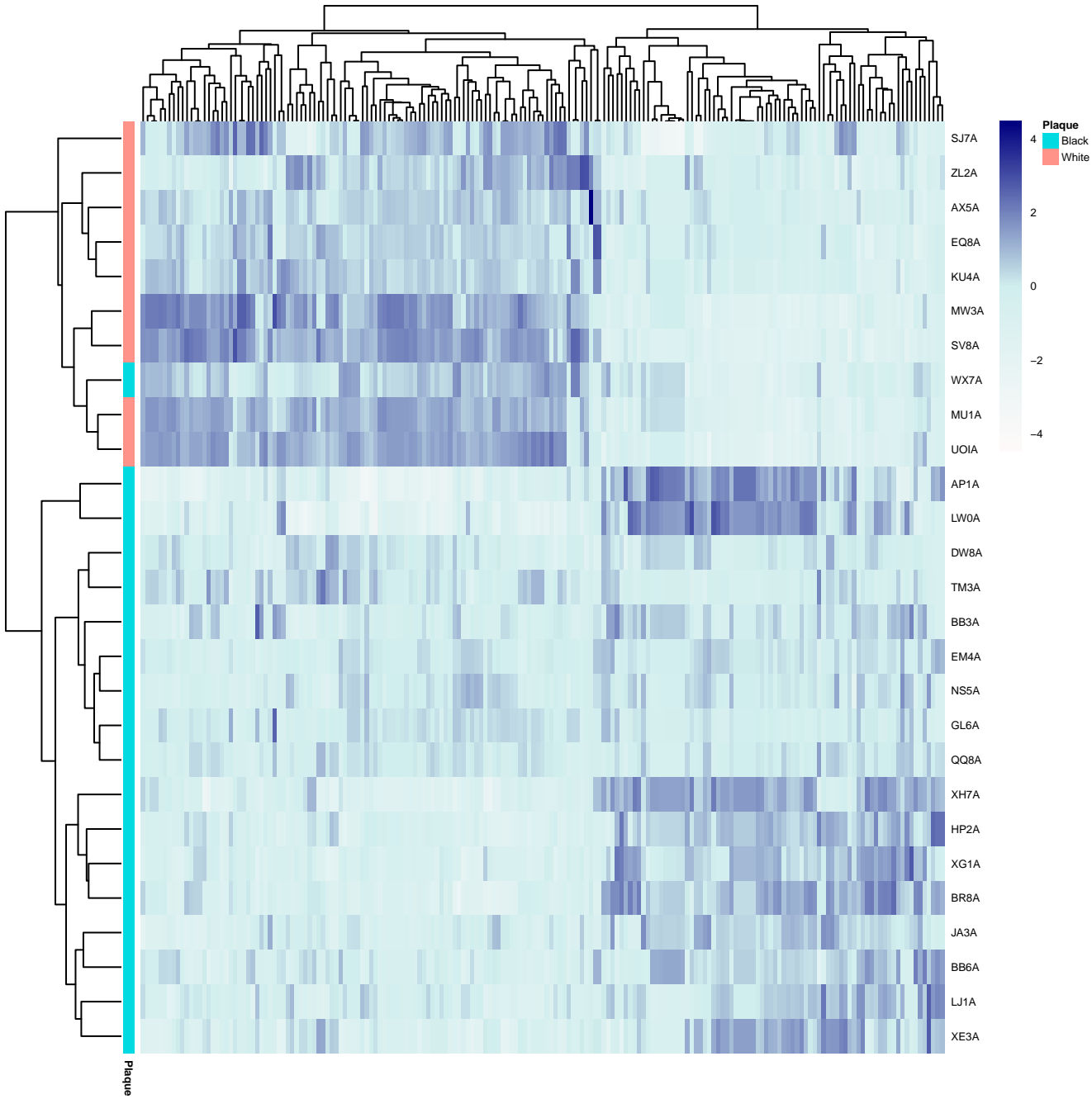

Supplement: Supplementary file 4 — Supplementary Figure 3. [file 41598_2020_72460_MOESM4_ESM.pdf]
