# Supplementary material for: Dental black plaque: metagenomic characterization and comparative analysis with white-plaque
Source: Sci Rep. 2020 Sep 29;10:15962. doi: 10.1038/s41598-020-72460-2 (PMC7525459; doi:10.1038/s41598-020-72460-2)
Supplement: Supplementary file 7 — Supplementary Legends. [file 41598_2020_72460_MOESM7_ESM.docx]

Supplementary Figure 1: The graphs present a visualization of the Jaccard qualitative distance and Bray Curtis quantitative distance showing differences between BS and white-plaque samples.

Supplementary Figure 2: Hierarchical clustering of samples according to the abundance of COG pathways.

Supplementary Figure 3: Hierarchical clustering of samples according to the abundance of Metacyc pathways.

Supplementary Table 1: Difference in relative abundance of 42 genera between black and white plaque samples, statistical significant taxa are shown in bold. P-values after Kruskal Wallis test with FDR correction.

Supplementary Table 2: The relative abundances of COG functional categories in the BS microbiome as compared to the white-plaque microbiome.

Supplementary Table 3: The Metacyc pathways identified in the BS microbiome.
